# Gel-Based Luminescent Conductive Materials and Their Applications in Biosensors and Bioelectronics

**DOI:** 10.3390/ma14226759

**Published:** 2021-11-10

**Authors:** Jiajin Qi, Gongmeiyue Su, Zhao Li

**Affiliations:** 1 Institute of Engineering Medicine, Beijing Institute of Technology, Beijing 100081, China; 3220201938@bit.edu.cn (J.Q.); gongmeiyue.su@bit.edu.cn (G.S.); 2 Beijing Key Laboratory for Separation and Analysis in Biomedicine and Pharmaceuticals, Beijing Institute of Technology, Beijing 100081, China

**Keywords:** gel-based materials, luminescence, conductivity, applications, biosensors, bioelectronics

## Abstract

The gel is an ideal platform for fabricating materials for bio-related applications due to its good biocompatibility, adjustable mechanical strength, and flexible and diversified functionalization. In recent decades, gel-based luminescent conductive materials that possess additional luminescence and conductivity simultaneously advanced applications in biosensors and bioelectronics. Herein, a comprehensive overview of gel-based luminescent conductive materials is summarized in this review. Gel-based luminescent conductive materials are firstly outlined, highlighting their fabrication methods, network structures, and functions. Then, their applications in biosensors and bioelectronics fields are illustrated. Finally, challenges and future perspectives of this emerging field are discussed with the hope of inspire additional ideas.

## 1. Introduction

With the increasing concern about health by people and large development in science, technology, and medicine, biosensors and bioelectronics became attractive research fields. Biosensors are devices used to detect biological substances through convert biological information into detectable signals [1,2,3]. Bioelectronics establish the connection between electronic device and the biological body and enable the device to capture and detect physiological signals, so that the biological condition of the biological body can be detected and evaluated [4,5,6,7]. Biosensors and bioelectronics experienced tremendous development in the past few decades, mainly due to the application of flexible materials in the fields. Different from traditional rigid biosensors and bioelectronics, flexible ones can establish tighter coupling and better compatibility with soft and dynamically deformed biological surfaces or internal organs, which makes them ideal candidates in monitoring and treatment of diseases, human body movements, and health indexes [8,9,10,11]. Currently, flexible biosensors and bioelectronics were widely used in the biomedicine, including biodetection sensors for biomarker detection and wearable bioelectronics devices for monitoring vital signs or capturing epidermal energy [12,13,14,15]. As the emerging frontier fields, it brings more requirements to the related materials, including performance and function requirements. On one hand, biosensors and wearable electronic devices are primarily applied in biomedical related areas, which require the materials with excellent biocompatibility and tunable mechanical properties [16,17,18]. On the other hand, the devices are required to convert the response signal into information that people can easily understand and directly observe.

The gel is an attractive material for research in recent years due to its unique characteristics such as outstanding biocompatibility, adjustable mechanical properties, quasi-liquid-solid behavior, scalability, and void structure [19,20]. In addition, functional materials can be facilely introduced into the gel network to endow it with corresponding functions. On the other hand, for the function requirement about signals, luminescent and electrical signals are two ideal candidates. The light-emitting process can convert the response into a light signal which owns advantages of high sensitivity and signal-to-noise ratio (SNR), fast response, easy detection, and pollution free [21]. The conductivity is one of the electric signals that can convert the response into resistance or conductivity change and has the superiorities of high selectivity and accuracy, good stability, easy to control, and high-familiarity [22,23].

Introducing luminescence or conduction function into gel matrix can afforded functional gel-based materials with corresponding characteristics. These functional materials were widely applied in numerous areas. For example, luminescent gels and conductive gels, which are widely concerned by people, were extensively used in sensors, bioelectronics, biomedicine, human-computer interaction, and soft robotics [24,25]. However, the singularity of the function in the gels restricts their further applications in many high-tech areas because many of the areas require the materials with multiple functions. Gel-based materials integrating the functions of luminescence and conductivity simultaneously can show both features of luminescence and conduction and in many cases have unexpected characteriastics due to the synergistic effect. The function versatility makes the material closer to the application demands in high-tech areas such as biosensors and bioelectronics. Therefore, the gel-based luminescent conductive material as an emerging advanced functional material has many superior characters and has made great progress in biosensors and bioelectronics recently.

**Figure 1:**
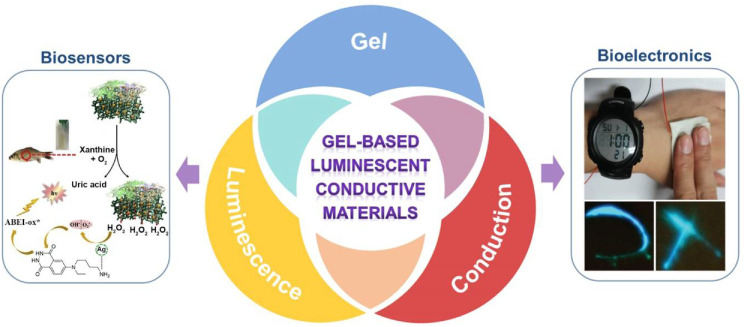
Summary of gel-based luminescent conductive materials and their applications in biosensors and bioelectronics. Figure of application example in biosensors was adapted with permission [26]; copyright 2019, Elsevier Ltd. Figure of application example in bioelectronics was adapted with permission [27]; copyright 2019, Wiley–VCH Verlag GmbH & Co. KGaA, Weinheim.

The research of gel-based luminescent conductive materials and bio-applications of them involves multidisciplinary knowledge and technologies. The interdisciplinary research can give birth to new direction and provide opportunities for more advanced applications. Although the field of gel-based luminescent conductive materials was developed rapidly in recent years, the periodical summary of it is lacking. An overview of this field can not only provide researchers a comprehensive understanding about it and motivate new ideas, but also showcase the scientific thought about interdisciplinary research. Therefore, herein a review about recent progress on gel-based conductive luminescent materials and their applications in biosensing and bioelectronics will be provided (Figure 1). Firstly, an overview of gels, luminescent materials, and conductive materials is provided. Then, gel-based luminescent conductive materials, including luminescent conductive gels and luminescent conductive gel composites, are summarized and reviewed. The preparation strategies, network structures, and performances about functions of luminescence and conductivity are highlighted. Subsequently, the application of the gel-based luminescent conductive materials in the areas of biosensing and bioelectronics is explained. Finally, current challenges and future prospects of this field are given, aiming to bring new insights into the development and commercial applications of biosensing and bioelectronic products.

## 2. Gels, Luminescent Materials, and Conductive Materials

### 2.1. Gels

The gel is a three-dimensional (3D) network filled with a dispersion medium in a matrix, and it is considered to be intermediate between liquid and solid. So far, various gels were developed and there are many classification standards to categorize them. For example, according to the dispersion medium inside the gel matrix, it can be classified as hydrogel, organic gel, aerogel, and xerogel. According to the constitution, gels can be divided into the macromolecular gel and supramolecular gel. The main constitution of the former is covalently formed macromolecules, while the major constituent of the latter is low molecular-weight gelators (LMWGs). Moreover, the gel can form through either physical or chemical bonds, indicating that the gels cross-linking by noncovalent physical bonds are physical gels, whereas crosslinking by covalent chemical bonds refers to chemical gels. In addition, gels can also be classified according to their size. The gel in macroscopic size is called macroscopic or bulk gel, and the gel with size in the micron or nanoscale is known as nano-micro gel [28,29,30,31,32,33].

### 2.2. Luminescent Materials

Luminescence mainly refers to the process of absorbing external energy inside an object at the ground state, reaching to the excited state, and finally, releasing the energy through light emission to get back to the ground state. According to the kind of energy absorbed, luminescence can be divided into photoluminescence (PL), electroluminescence (EL), chemiluminescence (CL), and sonoluminescence (SL). PL is a phenomenon in which an object emits light upon irradiation by ultraviolet light or visible light [34]. EL is the phenomenon of luminescence when an object passes through an electric current or is in an electric field [35]. CL refers to the luminescence phenomenon that accompanies the generation of light in the process of chemical reactions. Electrochemiluminescence (ECL) is one of a representative type of CL. It produces luminescence by applying a certain electrical signal to a chemical system containing chemiluminescent substances by electrodes [36,37]. SL occurs in liquids and refers to a kind of “sonic cavitation” phenomenon in the liquid when the liquid is subjected to strong sound waves. Specifically, bubbles are generated in the liquid, and the bubbles are instantly reduced to a tiny volume. During the process, they will emit flashes and release a large amount of heat [38].

Luminescent materials refer to functional materials that convert various forms of energy absorbed from the outside into light radiation. They usually emit visible light but can also be ultraviolet and infrared light. There are many types of luminescent materials, such as organic luminescent materials, transition metal complexes, luminescent nanomaterials, and zinc sulfide-based materials.

Organic luminescent materials can have luminescent pathways of photoluminescence, electroluminescence, chemiluminescence and electrochemiluminescence, in which PL organic compounds are in the majority. Interestingly, some organic PL materials can only emit light in a dilute solution. Once the concentration of the solution increases to accumulate the molecules, their luminescence will weaken or even disappear entirely. This phenomenon is called aggregation-caused quenching (ACQ) [39]. On the contrary, some organic molecules emit weakly in low-concentration solutions, but their PL is significantly enhanced after aggregation. This phenomenon is called aggregation-induced emission (AIE) [40]. PL materials with these phenomena or behaviors may endow their constructed materials with more diverse functions and stimuli-responsiveness. Currently, organic luminescence materials are widely used and are among the most critical vital materials in various application areas, such as fluorescent coatings, flexible optoelectronic devices, biological probes, information-related areas, and environmental protection. Luminol is one of the most commonly used organic CL materials, which can be oxidized by peroxides under alkaline conditions and emit blue light at the same time [41,42].

Transition metal-organic complexes have the advantages of both inorganic and organic compounds, which drew much attention in the field of luminescence. Iridium (Ir) complexes, ruthenium (Ru) complexes, and lanthanide complexes are typical examples of the metal-organic complexes [43,44,45]. Ir complexes are luminous bodies with good second- and third-order nonlinear optical responses. They have excellent properties, such as high luminescence quantum yield, large Stokes shift, and long luminescence lifetime [46]. Ru complexes are six-coordinate octahedral configuration complexes, a triplet luminophore with a long luminescence lifetime, long emission wavelength, and low cytotoxicity [47]. Lanthanum ion complexes are a kind of critical fluorescent material with unique metal-controlled photoluminescence behavior due to its particular electronic structure, high intensity, and high purity [48,49].

Fluorescent nanoparticles refer to nanoparticles or nanocrystals with less than 100 nm diameter that can emit fluorescence [50]. The present fluorescent nanoparticles mainly include quantum dots (QDs), carbon dots (CDs), metal nanoclusters (NCs), and perovskite nanocrystals (PNCs), and fluorescent dye-doped silica nanoparticles (DSNPs).

QDs with unique optical and electrical characteristics are semiconductor particles with nanostructure. Optoelectronic properties of QDs are related to the size, shape, and quantum physics of the particles [51]. CDs are composed of spherical-like carbon nanoparticles below 10 nm and are a new type of nano-carbon material with fluorescent properties [52,53]. NCs are composed of several to a few hundred atoms. Its size is between atoms and nanoparticles. NCs have different characteristics from metal nanoparticles, such as discrete electronic energy levels, high quantum yields, tunable fluorescence emission and good photostability. [54,55,56,57,58]. PNCs is a unique structure of perovskite, their size is on the order of nanometers, and the quantum effect is significant [59]. DSNPs are composite luminescent nanomaterials in which fluorescent dyes are doped into nano-silica [60].

Zinc sulfide (ZnS) is a vital semiconductor material of group II-VI with good optical and electrical properties, which can be applied in light-emitting diodes, optoelectronic devices, sensors, lasers, and other fields. ZnS alone is difficult to produce the stable and excellent performance of luminescence. Indeed, the performance of ZnS nanomaterials can be significantly improved through doping modification. The current doping mainly includes rare earth elements, transition metal elements, and other elements, such as zinc sulfide doped copper (ZnS:Cu) and zinc sulfide doped manganese (ZnS:Mn) [61,62].

### 2.3. Conductive Materials

Conductive materials are the substance that can provide conductivity under the action of an electric field. So far, various conductive materials were developed, mainly including conductive nanomaterials, conductive polymers, ionic conductors, and low-molecular weight organic compounds with the conjugated structure.

Ionic conductors conduct electricity through the directional movement of ions. Ionic conductors cannot complete the conductive task independently, and often need to be used in connection with electronic conductors. Usually, inorganic salt ions are dissolved in the solution or doped into materials containing the solution (such as hydrogel) to prepare the ion conductor. Most of the ions are free in the solution, namely free ions. In some cases, ions have interactions with material matrix. For example, some metal ions can form coordination bonds with gel network chain and act as both physical cross-linking points and conductive species [63,64].

Ionic liquids (ILs) are a special kind of ion conductor, which are liquids composed entirely of ions. It has the characteristics of high conductivity, low vapor pressure, and good stability. In addition, the melting point of ILs is relatively low, usually below 100 °C [65,66,67]. Deep eutectic solvents (DES) are composed of two or more clean and safe ingredients (urea, choline chloride, etc.) through hydrogen bond interaction. Deep eutectic solvents usually remain liquid below 100 °C. They have many similarities with ionic liquids but are cheaper and safer than ionic liquids [68,69].

Some low-molecular weight conjugated organic compounds can also exhibit conductivity in supramolecular gels. These compounds can assemble to form fibers through noncovalent interactions such as π-π stacking, donor-acceptor interaction, hydrogen bond, electrostatic interaction, and van der Waals interaction. The fibers can form network through further assemble and intertwine. Electrons can transport through the π-conjugated fiber chain, which endow the material with conduction [70,71,72].

Conductive polymers are organic electronically conjugated macromolecules characterized by their ability to conduct electrons. They transport electrons through π-conjugated chains and “doping” processes involving chemical or electrochemical methods. Polyaniline (PANi), polythiophene, polypyrrole (PPy), and poly(3,4-ethylenedioxythiophene): polystyrene sulfonate (PEDOT:PSS) are typical conductive polymers. PANi, a chemical or electrochemical polymerized monomer aniline substance, are commonly used conductive polymers to construct soft conductive materials. It has the advantages of easy synthesis, antibacterial property, high conductivity, and promotion of cell proliferation and differentiation [73,74].

Conductive nanomaterials are another large group of conductive materials. They are mainly divided into two categories, carbon-based nanomaterials, and metal-based nanoparticles. Carbon-based nanomaterials mainly include graphene, carbon black, carbon fibers (CFs), fullerene, and carbon nanotubes (CNTs). Carbon-based nanomaterials are considered one of the most promising conductive materials due to their unique high conductivity, environmental stability, and low production cost. Among the above-mentioned carbon-based nanomaterials, graphene is a two-dimensional carbon nanomaterial composed of a single layer of carbon atoms with an sp^2^ hybrid hydrocarbon skeleton, which has superconductivity, high surface area, outstanding thermal conductivity, and excellent mechanical strength. Thus, it is an excellent carbon-based conductive dopant to be widely applied in soft materials for biosensing and bioelectronics [75,76]. Metal-based nanoparticles are nano-scale particles, and their optical properties and electrical conductivity are affected by the size. Metal-based nanomaterials have a high electrical conductivity of bulk metals, and have the properties of nanomaterials (magnetic properties, catalytic properties, and antibacterial properties), making them have potential applications in biomedicine. Au nanoparticles (Au NPs), Au nanoclusters (Au NCs), Ag nanofibers (Ag NFs), and Ag nanowires (Ag NWs) are typical metal-based nanoparticles usually used in soft conductive materials. In particular, Au NPs are critical metal nanoparticles with unique conductive, optical, and magnetic properties, ease synthetic procedure, high stability, and good biocompatibility.

## 3. Gel-Based Luminescent Conductive Materials

The gel is a remarkably flexible material. In recent years, due to the good biocompatibility, flexibility, stretchability, and functionality, it was always at the forefront of developing smart materials. Gel-based luminescent conductive materials are an emerging type of multifunctional flexible materials with both luminescent and conductive properties, which is an ideal choice for wearable electronic devices, sensors, soft robotics, and many other high-tech field applications [77,78,79,80]. This section will review gel-based luminescent conductive materials, focusing on their preparation, constitution and network structure, and properties. Two kinds of materials are involved. One is the luminescent conductive gel, which is a gel with both luminescent and conductive properties. The other is gel composite with luminescent and conductive properties, composed of luminous or conductive gel combining with flexible materials such as elastomers.

### 3.1. Luminescent Conductive Gels

Luminescent conductive gels are generally prepared by introducing conductive materials and luminescent materials into the gel matrix. Different synthetic methods were developed to prepare luminescent conductive gels.

**Figure 2:**
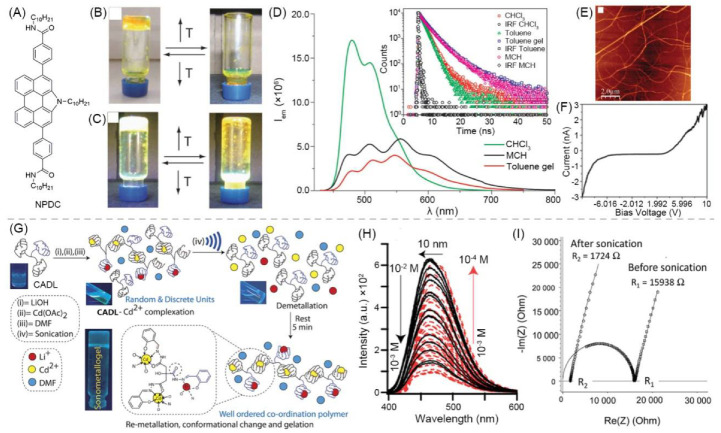
(**A**) Chemical structure of NPDC. Photographs of gel-to-sol transition of NPSC-based organogel without (**B**) and with (**C**) 360 nm UV illumination. (**D**) PL spectra of NPDC under different conditions (*λ*_ex_ = 420 nm) with inset showing fluorescence lifetime-decay profiles. (**E**) AFM image of aggregations of NPDC. (**F**) CAFM image of toluene gel of NPDC onto HOPG as surface. Adapted with permission [81]; copyright 2013, Royal Society of Chemistry. (**G**) Schematics of fabrication procedure of sonometallogel from CADL and metal ions along with fluorescent photographs. (**H**) PL spectra showing dilution of sonometallogel to demonstrate presence of AIE and ACQ phenomena. (**I**) Experimental Nyquist impedance diagrams before and after sonication. Adapted with permission [82]; copyright 2017, Royal Society of Chemistry.

Luminescent materials and conductive materials can also be components of the gel network. Ajayaghosh and Sánchez et al. reported that *N*-annulated perylenedicarboxamide (NPDC) (Figure 2A) self-assembled in toluene to form columnar aggregate fibers. Then, the fibers bound to form an organic gel. Fluorescent property of NPDC endowed the gel with luminescence behavior. The gel can convert between sol and gel under temperature changes, and emit light green fluorescence under 360 nm ultraviolet (UV) light (Figure 2B,C). The emission spectrum of the gel covers the visible spectrum and shows maximum emission intensity at about 546 nm (Figure 2D). Atomic force microscopy (AFM) imaging revealed the gel is composed of fine thread-like fibers (Figure 2E). NPDC is low-molecular weight conjugated organic compounds, which can form a π-conjugated chain through π-π stacking. Electrons are transported through the π-conjugated chain, making the gel conductive. The electrical conductivity of the gel is good, and its electrical conductivity measured by Four-Probe Conductivity (FPC) is 1.92 × 10^−4^ S·m^−1^ (Figure 2F) [81].

Introducing metal ions to coassemble with LMWGs can also affords supramolecular luminescent conductive gel. Dubey et al. used a citric acid derived ligand (CADL), LiOH, and Cd(OAc)_2_ to synthesize a fluorescent metal gel with multistimuli responsiveness through ultrasound induction in *N*,*N*-dimethylformamide (DMF). At the beginning, Cd^2+^ and CADL complexed without forming a gel. After ultrasonic treatment, the Cd^2+^ ions were demetalized and recomplexed, eventually leading to gelation (Figure 2G). The gel emits blue light when irradiated by UV light. It emits light with AIE and ACQ phenomena. The fluorescence intensity decreases when the gel is diluted from 10^−2^ M to 10^−3^ M, and the fluorescence intensity increases from 10^−3^ to 10^−4^ M (Figure 2H). The gel conducts electricity through the directional movement of Li^+^ and Cd^2+^ in the network. The conductivity of the gel itself is poor (4.5 × 10^−3^ S·m^−1^), but it can be effectively increased by about ten times after ultrasonication (4.06 × 10^−2^ S·m^−1^) (Figure 2I). This is because the inside of the gel becomes more organized or orderly after ultrasonication, which increases the Li^+^ mobility [82].

Table 1 summarized that some typical luminescent conductive gels are composed of self-assembled functional low molecular-weight gelators or low molecular-weight gelators with ions constitute gel matrix.

**Figure 3:**
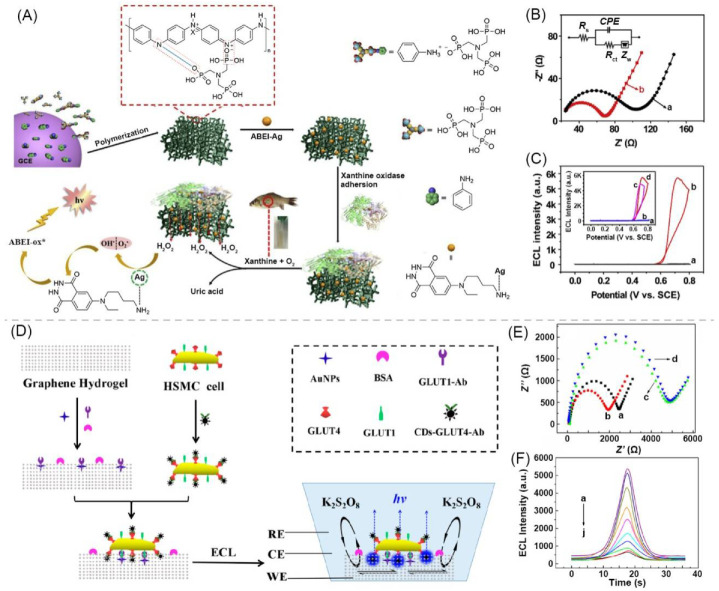
(**A**) Schematic of preparation process of PAni polymeric hydrogel-based ABEI–Ag@PAni–ATMP ECL biosensor for xanthine. (**B**) EIS of Ani-ATMP modified GCE (a) and PAni–ATMP modified GCE (b). (**C**) ECL-potential curve of ABEI–Ag@PAni–ATMP in presence of 20 μM H_2_O_2_ with a scan rate of 50 mV·s^−1^. Adapted with permission [26]; copyright 2019, Elsevier Ltd. (**D**) Schematic of fabrication process of graphene hydrogel-based ECL cytosensor. (**E**) EIS spectra of GH (a), GH/AuNPs (b), GH/AuNPs/GLUT1–Ab/BSA (c), and GH/AuNPs/GLUT1–Ab/BSA/cell@CDs–GLUT4–Ab (d). (**F**) ECL curves obtained with different recombinant GLUT4 concentrations (0 to 4.5 ng·mL^−1^ from a to j). Adapted with permission [83]; copyright 2019, American Chemical Society.

The polymer-based gel can be used as a matrix to construct luminescent conductive gels. Li and Shan et al. designed an ECL hydrogel for xanthine detection. Firstly, a conductive polymer hydrogel of polyaniline (PAni) (PAni–ATMP) was prepared from aniline (Ani), amino trimethylene phosphonic acid (ATMP), and (NH_4_)_2_S_2_O_8_. Then, the *N*-(aminobutyl)-*N*-(ethylisoluminol) functionalized silver nanoparticles (ABEI–Ag) were immobilized on the hydrogel (ABEI–Ag@PAni–ATMP) by ATMP. Finally, the gel was combined with xanthine oxidase (XOD). The electrical conductivity of the gel was provided by PAni. When xanthine acts on the hydrogel with an electric field, H_2_O_2_ is decomposed by electrochemically reacting with ABEI–Ag, and an ECL signal occurs (Figure 3A). The Nyquist plot was measured with Ani–ATMP and PAni–ATMP modified glassy carbon electrodes (GCE). Compared with the semicircle diameter of Ani–ATMP, the semicircle diameter of PAni–ATMP modified GCE was significantly reduced, indicating that the gel has excellent conductivity (Figure 3B). In the presence of 20 μM H_2_O_2_ on ABEI–Ag@PAni–ATMP gel, a clear ECL signal was observed, and the ECL signal reached about 6000 au., the ECL peak was located at about 0.7 V (Figure 3C) [26].

There are also some other typical examples of luminescent conductive gels with a polymer-based network as the matrix. The information about them is also summarized in Table 1.

Carbon-based nanomaterials can also be used as a matrix to construct luminescent conductive gels. Jin and Chen et al. prepared an ECL gel based on graphene hydrogel (GH). The preparation scheme is shown in Figure 3D. Au NPs modified with glucose transporter 1 antibody (GLUT1–Ab) and bovine serum albumin (BSA) were immobilized on GH. Human skeletal muscle cells (HSMC) containing GLUT1 and GLUT4 on the surface are labeled with GLUT4–Ab-functionalized carbon dots (CDs–GLUT4–Ab) by GLUT4. After the labelled cells were fixed on the GH-based electrode, a gel with ECL is prepared (GH/AuNPs/GLUT1–Ab/BSA/cell@CDs–GLUT4–Ab). When an electrochemical reaction occurred, the co-reactant K_2_S_2_O_8_ diffused through the GH and reacted with the CD on the bottom surface of the HSMC to generate an ECL signal (Figure 3D). GH/AuNPs/GLUT1–Ab/BSA/cell@CDs–GLUT4–Ab is conductive (Figure 3E). As the concentration of GLUT4 increased, the ECL peak value continued to decrease (Figure 3F) [83].

In addition, some representative examples, which are some luminescent conductive supramolecular gels constructed from nanomaterials, are summarized in Table 1.

Besides luminescent conductive gels, some photoluminescent supramolecular gels can respond to the electric field. The medium of the gel is liquid crystal, which has certain responsiveness to the electric field. Networks of the gels are formed through the self-assembly of luminescent low-molecular-weight gelators. The liquid crystal can be aligned by applying the electric field and changing the transmittance of the gel. Under the action of an electric field, the optical behavior, such as fluorescence intensity [84], switching of optical transmittance [85], and optical contrast [86], of the luminescent gel can change.

### 3.2. Luminescent Conductive Gel Composites

**Figure 4:**
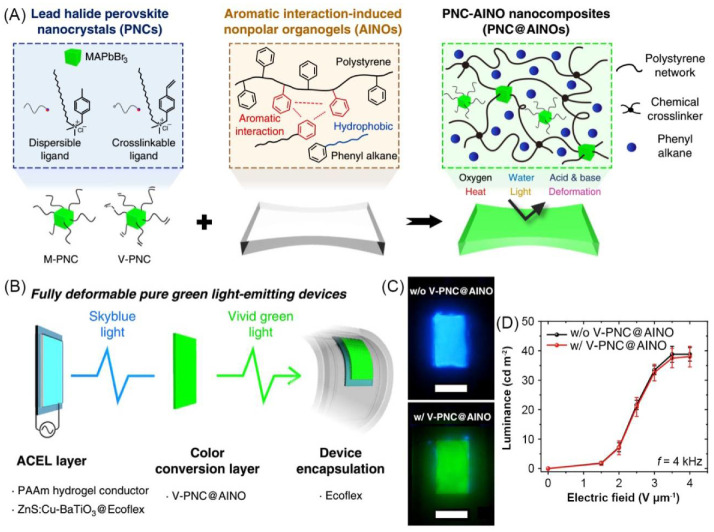
(**A**) Schematic of structure of green luminescent perovskite nanocomposite gel. (**B**) Schematic of design of the organogel/Ecoflex/organogel composite-based light-emitting device. (**C**) Photographs of emission color from ACEL layer without (upper) and with V-PNC@AINO layer (lower). Scale bar = 1 cm. (**D**) Applied AC electric field dependence of Luminance of devices without and with V-PNC@AINO layer. Adapted under terms of Creative Commons Attribution 4.0 International License (CC BY 4.0) (https://creativecommons.org/licenses/by/4.0/, accessed on 3 September 2021) [116]; copyright 2020, authors, published by Springer Nature.

The luminescent conductive gel composite is a material composed of multiple layers of materials with the participation of gel. It is mainly composed of a conductive layer and a luminescent layer [99,112,113,114,115]. Park et al. combined methylammonium lead bromide perovskite nanocrystals (MAPbBr_3_ PNCs) with aromatic interaction-induced nonpolar organogels (AINOs) through physical or chemical interactions to prepare luminescent green nanocomposite gels (PNC@AINOs). Among them, the one that bound MAPbBr_3_ PNCs to AINOs through physical interaction is M-PNC@AINOs, and the one that bound chemically is V-PNC@AINOs. PNC@AINO gel can emit bright green light under UV light (Figure 4A) originating from PNCs. V-PNC@AINO was used to fabricate a gel composite which was mainly composed of three layers. The first layer was a LiCl containing polyacrylamide (PAAm) hydrogel. The second layer was an EL elastomeric layer composed of ZnS:Cu-BaTiO_3_ and Ecoflex. The above two layer constituted a stretchable alternating current (AC) electroluminescence (ACEL) layer. The third layer was the V-PNC@AINO color conversion layer (Figure 4B). The ACEL layer can emit bright sky-blue light under the action of an AC electric field. When an AC electric field is applied to the device, the blue light emitted by the ACEL layer excites V–PNC@AINO to emit bright green light through photoluminescence (Figure 4C). The composite device has very little loss of brightness during the color conversion process. Regardless of the applied electric field, the color conversion efficiency remains around 97% (Figure 4D) [116].

For another example, Fan and Zhi et al. reported a self-healable electroluminescence gel composite device with a sandwich structure. The top and bottom layers are composed of self-healable conductive polyacrylic acid hydrogel, and the middle layer is composed of self-healable phosphor polyurethane. The device emitted blue light after being energized. Because of the self-healing property, the luminescence performance of the devices can be recovered with high healing efficiency. [117]. Yuan et al. reported an electroluminescence gel composite device with the same sandwich structure. LiCl/agar/polyacrylamide double network hydrogel gel was used as the top and bottom electrodes. The electroluminescent emissive middle layer was composed of polydimethylsiloxane (PDMS) and ZnS:Cu. The device emits blue light when it is energized. The device made of the ion hydrogel as the conductive layer could obtain better luminous brightness than the indium tin oxide (ITO) as the conductive layer [118].

Typical examples of some luminescent conductive gel composites constructed by combining gel with other materials are summarized in Table 2.

## 4. Applications in Biosensors and Bioelectronics

Gel-based luminescent conductive materials are a combination of conductive materials, luminescent materials, and gels. They were widely applied in biology-related areas. In this section, the applications in the areas of biosensors and bioelectronics are reviewed.

### 4.1. Biosensors

A biosensor refers to a device that can convert the concentration information of biological substances into signals such as light and electricity. The development of high-sensitivity, fast response, convenient manipulation, and low-cost biosensors is important for modern biomedicine. Luminescent conductive gels with features of gels, luminescent and conductive materials are suitable platforms for biosensor development. Particularly, luminescent conductive gels with ECL are widely used as biosensors to detect biomolecules, cells, and microorganisms.

**Figure 5:**
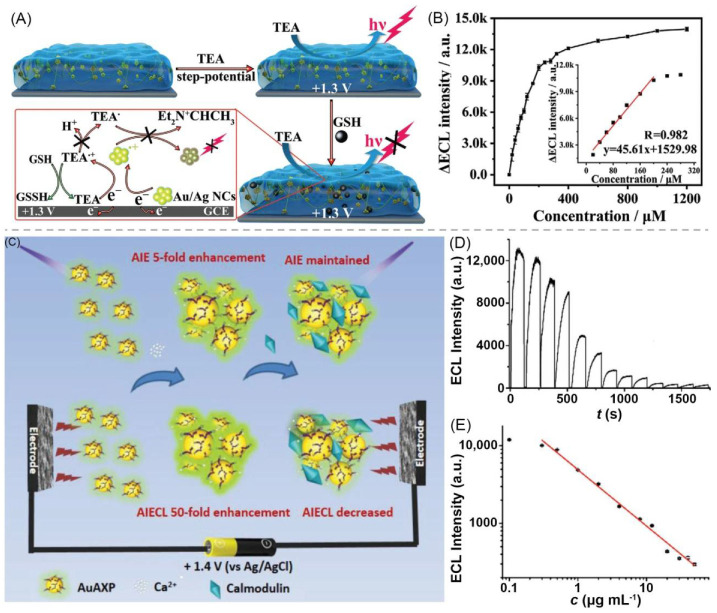
(**A**) Schematic of mechanism of GSH sensing by Au/Ag NCs@BSA hydrogel. (**B**) Calibration plot about ΔECL intensity as a function of GSH concentration with inset being linear relationship between ΔECL intensity and GSH concentration. Adapted with permission [97]; copyright 2020, Wiley–VCH Verlag GmbH & Co. KGaA, Weinheim. (**C**) Schematic of ECL calmodulin sensor based on bivalent cations-NCs hydrogel. (**D**) Relationship between ECL intensity and CaM concentration. (0–50 μg mL^−1^ from left to right) (**E**) ECL intensity as a function of CaM concentration. Adapted with permission [98]; copyright 2019, Wiley–VCH Verlag GmbH & Co. KGaA, Weinheim.

Guo et al. reported a hydrogel biosensor that can be used to detect glutathione (GSH). The hydrogel comprises BSA and Au/Ag alloy nanoclusters (Au/Ag NCs). The sensing system detects GSH through the decrease of ECL intensity in the presence of triethylamine (TEA). This mechanism is due to the reaction between GSH and TEA^•+^ radicals produced from electro oxidation of TEA, which inhibits the reaction between TEA^•+^ radicals and Au/Ag NCs, leading to the quenching of ECL (Figure 5A). The ECL intensity of the sensor decreases as the GSH concentration increases. When the GSH concentration is between 20 and 200 × 10^−6^ M, the ΔECL signal (ΔECL = *I*_0_ − *I*) has a linear relationship with the GSH concentration, and the correlation coefficient is 0.982. The detection limit is 8.7 × 10^−6^ M (Figure 5B). In addition to detecting GSH, the sensing system also has anti-biofouling and self-healing properties. These characteristics make it suitable for long-term biosensing applications [97]. Wang et al. reported a hydrogel biosensor that can be used to detect calmodulin (CaM). The gel network was formed by connecting Au AXP and Ca^2+^. The gel has both AIE and aggregation-induced electrochemiluminescence (AIECL) behaviors, and its AIECL signal is 10-times that of AIE (Figure 5C). CaM can specifically bind to the Ca^2+^ inside the gel to effectively regulate AIECL dynamics, and the ECL signal decreases with the increment of CaM concentration (Figure 5D). Within 0.3–50 µg·mL^−1^, the ECL intensity of the biosensor has a linear relationship with the CaM concentration. The detection limit of the sensor is 0.1 µg·mL^−1^ (Figure 5E) [98].

**Figure 6:**
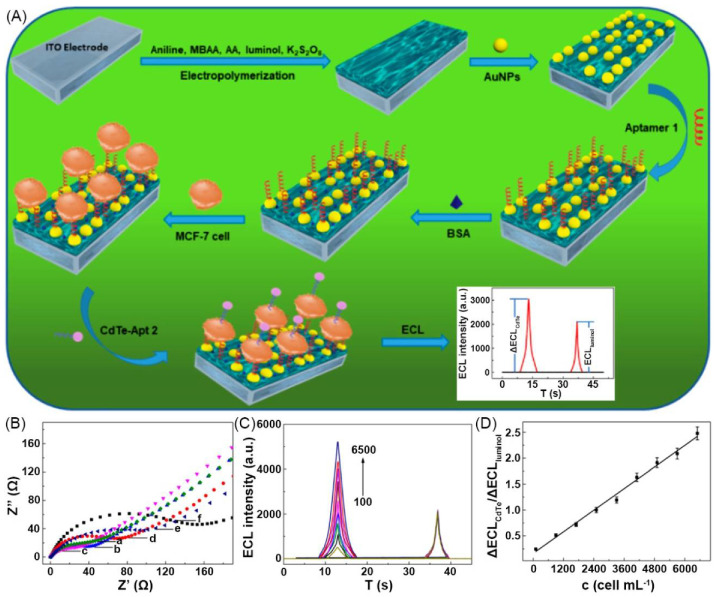
(**A**) Schematic of ratiometric ECL cytosensor working principle. (**B**) EIS characterization of sensing interfaces in 0.1 PBS (pH 7.4) with 0.1 KCl and 5.0 mM [Fe(CN)_6_] ^3–^^/4–^. (**C**) Time dependence of ECL intensity of CdTe nanoprobes with different MCF-7 cell concentrations (100–6500 cells·mL^−1^). (**D**) Calibration ΔECL_CdTe_/ΔECL_luminol_−cell concentration curve. Adapted with permission [96]; copyright 2019, American Chemical Society.

Ding and Luo et al. reported the construction of an ECL hydrogel cell sensor for detecting cancer cells. PAni-based conductive polymer hydrogel (CPH) was modified with aptamer-tagged Au NPs and was deposited on ITO electrodes. CdTe QDs tagged with the aptamer (CdTe-Apt 2) of cancer cells were used to label cancer cells. The aptamer part facilitated the capture of the cancer cells by the hydrogel electrode surface and the QD part acted as the detection signals. Luminol as another luminogen embedded in CPH was used as internal standards. The electrochemiluminescence of the formed ratiometric sensor system has double peaks, one is the signal of CdTe-Apt 2 on cancer cells and the other is the luminol signal as the internal standard (Figure 6A) to quantify cancer cells by comparing the sensitivity difference of the bimodal ECL signals with that of target analytes. The ECL intensity of CdTe QDs increases with the increment of cell concentration from 100–6500 cells mL^−1^, while the ECL intensity of luminol keeps unchanged (Figure 6C). The ratio of ΔECL_CdTe_ and ΔECL_luminol_ has a linear relationship with the concentration of target cells in the cell concentration range. The detection limit of the sensor is 80 cells mL^−1^ (Figure 6D) [96].

**Figure 7:**
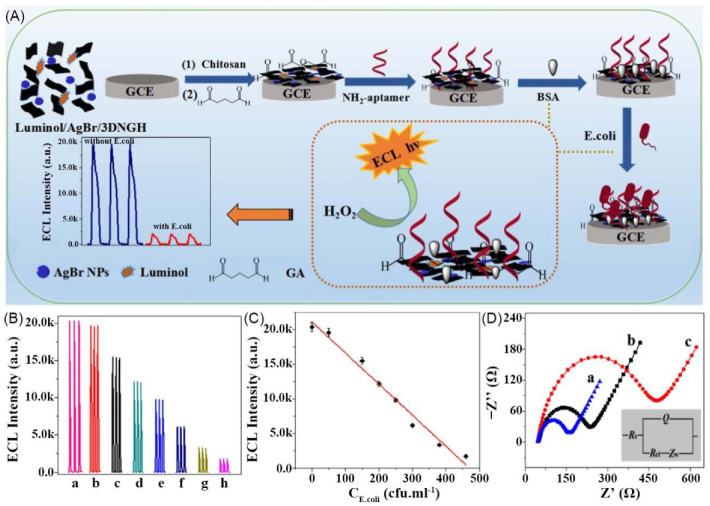
(**A**) Schematic of fabrication process of luminol/AgBr/3DNGH-based ECL E. coli biosensor. (**B**) ECL responses of BSA/aptamer/GA/CHIT/luminol/AgBr/3DNGH/GCE in 0.1 M PBS (pH 8) with different concentrations of E. coli. (**C**) Calibration curve for E. coli detection. (**D**) EIS of GA/CHIT/luminol/AgBr/3DNGH/GCE (a), BSA/aptamer/GA/CHIT/luminol/AgBr/3DNGH/GCE nanocomposites before (b) and after (c) E. coli reaction. Adapted with permission [94]; copyright 2017, Elsevier Ltd.

For the microorganism sensing, Wang et al. reported an ECL Escherichia coli (E. coli) aptasensor which was prepared by luminol, AgBr NPs, 3D nitrogen-doped GH (3DNGH), and amine-functionalized E. coli aptamer (NH_2_-aptamer) (Figure 7A). This sensor can respond E. coli by decreasing the ECL intensity. As the concentration of E. coli increases, the ECL intensity gradually decreases (Figure 7B). The sensor has good performance with the linear sensing range of 0.5–500 cfu·mL^−1^ and the detection limit of 0.17 cfu·mL^−1^ (Figure 7C) [94].

### 4.2. Bioelectronics

In recent years, bioelectronics has gradually changed human-computer interaction, such as wearable electronic devices, implantable electronic devices, and human motion detection systems. Flexible bioelectronics can accurately respond to various stimuli in the environment in real-time and output in the form of electrical signals. Moreover, it plays a critical role in intelligent sports, software robots, biomedical monitoring, and diagnosis confirmation. Gel-based luminescent conductive materials combine the flexibility and good biocompatibility of gels with conductivity and fluorescence that can respond to environmental stimuli, making them have excellent application prospects in the field of flexible bioelectronics.

**Figure 8:**
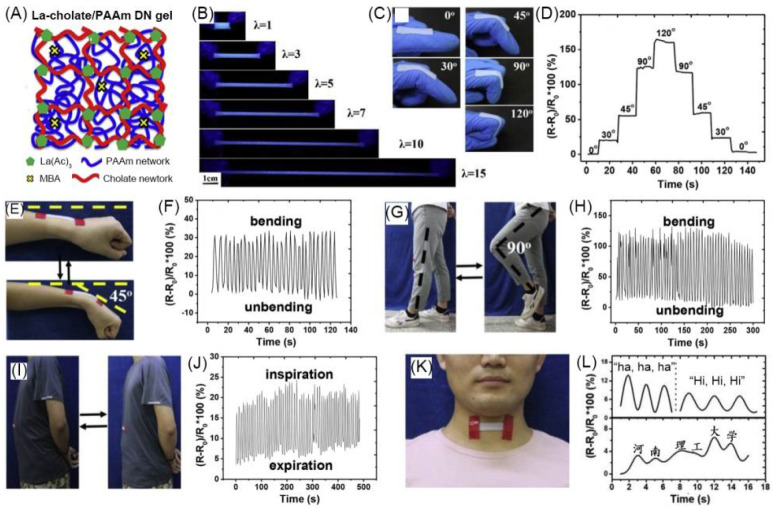
(**A**) Schematic of La-cholate/PAAm DN hydrogel network structure. (**B**) Fluorescent photographs of hydrogel under different strains. (**C**) Photographs show hydrogel adhered on a finger with different bending angles. (**D**) Change of resistance ratio of hydrogel with different bending angles. Photographs showing cyclic bending of wrist from 0 to 45° (**E**) and relative resistance ratio change (**F**). Photographs showing cyclic bending of knee from 0 to 90° (**G**) and relative resistance ratio change (**H**). (**I**,**J**) Recording breathing during inspiration and expiration. (**K**,**L**) Detection of vibration of human throat during speaking. Adapted with permission [110]; copyright 2019, Elsevier Ltd.

Chen et al. designed and fabricated a luminescent conductive La-cholate/PAAm double network (DN) hydrogel with La^3+^-cholate-based supramolecular network as the first network and chemically cross-linked polyacrylamide (PAAm) as the second network (Figure 8A). The hydrogel emits blue fluorescent light, and the emission intensity decreases as the stretched length increases (Figure 8B). The ionic conductivity of the gel is 0.3 S·m^−1^, and it decreases as the stretched length increases. The gel was used as a strain sensor to monitor human movements. The results showed that the DN hydrogel can detect the movement of fingers, wrists, and knees. When the finger attached by the gel was bent at different angles from 0 to 120°, the resistance ratio of the gel could be observed to increase and recover at different angles (Figure 8C,D). The gel could also show resistant signal changes under the cyclic wrist bending from 0° to 45° (Figure 8E) and the knee bending 0° to 90° (Figure 8G). In addition, La-cholate/PAAm DN hydrogel can also detect human breathing and speech. When the gel was applied to the abdomen, the gel sensor could clearly record the small changes in the abdomen during breathing (Figure 8I,J). At the same time, the gel sensor located in the throat can change the resistance according to the vibration of the throat when speaking (Figure 8K,L) [110].

**Figure 9:**
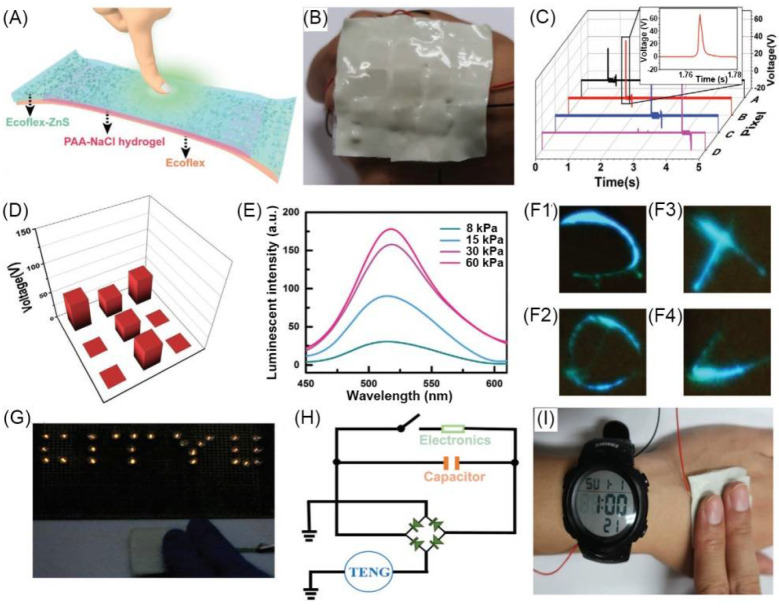
(**A**) Structural configuration of Ecoflex/hydrogel/Ecoflex gel composite-based smart skin device. (**B**) Photograph of the smart skin containing nine hydrogel pixels on back of a hand. (**C**) Electric signals at different pixels for detection of pressure location. (**D**) Voltage signals of nine pixels with a “T” shaped acrylic plate placed on skin. (**E**) Mechanoluminescent intensity of skin at various pressure magnitudes. (**F1–4**) Photographs of a real-time tracking of ML signals by external force. (**G**) Photograph of smart skin to power LEDs to demonstrate energy-harvesting property. (**H**) Equivalent circuit of a self-charging system to power an electronic watch (**I**). Adapted with permission [27]; copyright 2019, Wiley-VCH Verlag GmbH & Co. KGaA, Weinheim.

Zhi et al. developed a multifunctional wearable smart skin device with touch-sensing, exteroception-visualizing, and energy-harvesting capabilities. The device consisted of three layers of dielectric elastomer (Ecoflex), conductive polyacrylic acid (PAA) hydrogel containing NaCl, and Ecoflex-ZnS EL layer (Figure 9A). To study the function of smart skin, a 3 pixels × 3 pixels sensor array was made with every constituted block of a size of 1 × 1 cm. The sensor array was attached to the hand, and when a single pixel was pressed in chronological order, the corresponding voltage signal output could be observed (Figure 9B,C). When a T-shaped acrylic plate was placed on the smart skin pixel, the force of the T position could be effectively detected (Figure 9D). The luminescence of the smart skin can respond to changes in force, and the luminous intensity has a nonlinear positive correlation with the applied force (Figure 9E). In addition, by randomly drawing various shapes on the smart skin, the real-time luminescence signal of the skin could be immediately visualized (Figure 9F). The smart skin can also collect mechanical energy, which can light up the LED circuit by tapping the smart skin with hand (Figure 9G). The mechanical energy of the smart skin can be converted into electrical energy for continuous output and can charge the electronic watch (Figure 9I). Figure 9H is the equivalent circuit of this process [27].

## 5. Conclusions and Perspectives

The gel-based luminescent conductive material is a perfect combination of various properties such as adjustable mechanical strength, biocompatibility, luminescence, and conductivity. The synergistic collection of these properties means that gel-based luminescent conductive materials benefit from these properties in the application and have the advantage of synergistic properties. Compared with that of traditional rigid sensors and electronic devices, the flexibility, good mechanical properties, and biocompatibility of gel-based luminescent conductive materials enable them to be used in nonplanar and various complex biological environments. Moreover, gel-based luminescent conductive materials have the advantages of conductive functions in sensing and electronic devices, such as easy control, high accuracy, and mathematics of signals. Also, they have the advantages of high sensitivity and wide linear range in the detection of luminescence functions. Meanwhile, dual-signal detection and comparison make them more reliable and accurate than single-signal sensors and electronic devices. Diverse gel-based luminescent conductive materials were developed due to the diversity of choices on gels, luminescent materials, and conductive materials. With excellent optoelectronic features and biocompatibility, the gel-based luminescent conductive materials have shown great potential in applications in biosensors and bioelectronics.

Although gel-based luminescent conductive materials have many excellent characteristics, there are still many challenges to be faced. Firstly, excellent mechanical properties are significant to wearable bioelectronics. The mechanical properties of current luminescent conductive gels need to be improved. Their mechanical properties can be increased by using advanced high-strength gel structures such as double network and nanocomposite network. Secondly, the biocompatibility of the gel-based luminescent conductive materials needs to be comprehensively evaluated in clinical application. Thirdly, although gel-based luminescent conductive materials were developed and applied in biosensors and bioelectronics, their luminescent and electrical properties are generally used independently. Therefore, it is necessary to design the gel network structure reasonably and intelligently at the microscope level (molecular level or aggregation level) to realize the synergistic effect of the two properties and significantly improve the material properties. Finally, although gel-based luminescent conductive materials were applied in the fields of biosensors and bioelectronics, their application is still in its infancy, and further research and development are needed to enable commercial applications.

Overall, gel-based luminescent conductive materials are anticipated to serve as a powerful platform for development of advanced biosensors and bioelectronics. It is hoped that this review could inspire more interest in this emerging field. In addition, the illustration of the research approach about this interdisciplinary field is hoped to showcase the thought to exploit new interdisciplinary fields.

## Figures and Tables

**Table 1:** Summary of information of typical luminescent conductive gels involved in this review.

No.	**Gel Type**	Network ^a^	Dispersing Medium ^b^	Conductive Species	Electrical Parameter	Luminescent Species	*λ*_ex_/*λ*_em_ (nm) ^c^	Application	Ref.
1	supramolecular bulk organogel	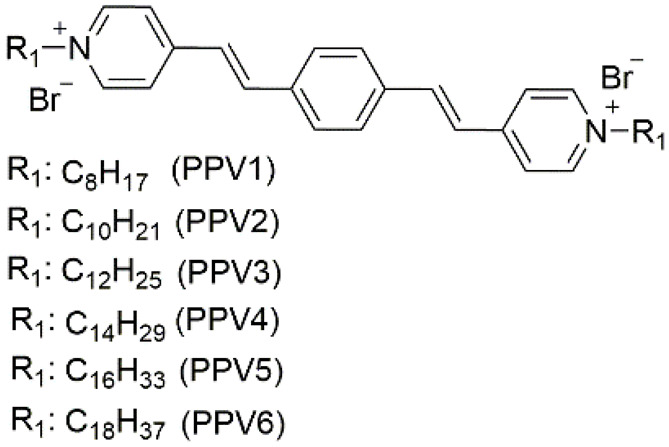	AN (PPV4), EG (PPV3–5), EtOH/H_2_O (PPV1–7), MeOH/H_2_O (PPV3–5), PrOH/H_2_O (PPV3–5), *t*-BuOH/H_2_O (PPV3–5)	PPV1–7	Resistance (Ω) (10V) (xerogel): 1.1 × 10^7^ (PPV1) 3.5 × 10^7^ (PPV2) 4.7 × 10^7^ (PPV3) 9.1 × 10^7^ (PPV4) 2.7 × 10^7^ (PPV5) 4.0 × 10^7^ (PPV6)	PPV1–7	470/~600 (PPV4) (EtOH/H_2_O)	-	[87]
2	supramolecular bulk organogel	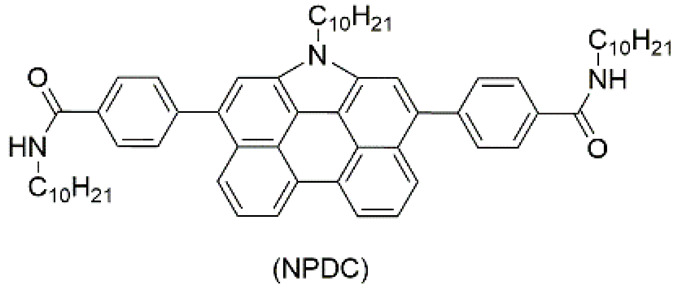	toluene	DNPC	Conductivity (S·cm^−1^): 2.16 × 10^−6^ (C-AFM), 1.92 × 10^−6^ (FPC)	DNPC	420/~546	-	[81]
3	supramolecular bulk organogel	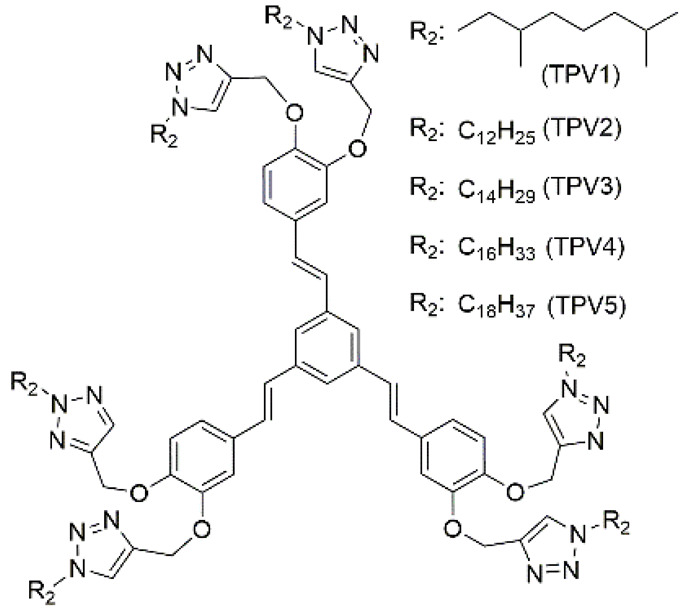	hexane, decane, dodecane, hexadecane	PPV1–5	Hole mobility (cm^2^·V^−1^·s^−1^) (80 V): 3.07 × 10^−3^ (TPV2); 3.19 × 10^−3^ (TPV3); 3.30 × 10^−3^ (TPV4); 3.47 × 10^−3^ (TPV5)	PPV1–5	-/~433	Fe^2+^ sensing, pollen grains imaging	[88]
4	supramolecular bulk xerogel	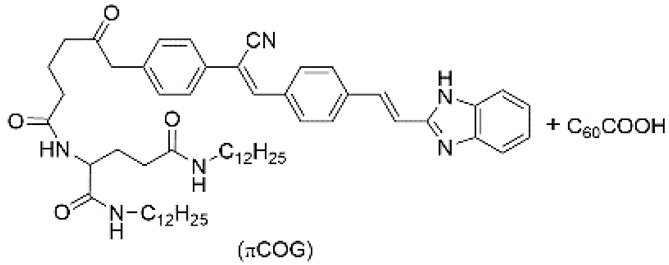	air	πCOG, C_60_COOH	Photocurrent (μA): ~4.5	πCOG	-/~530	-	[89]
5	supramolecular bulk organogel	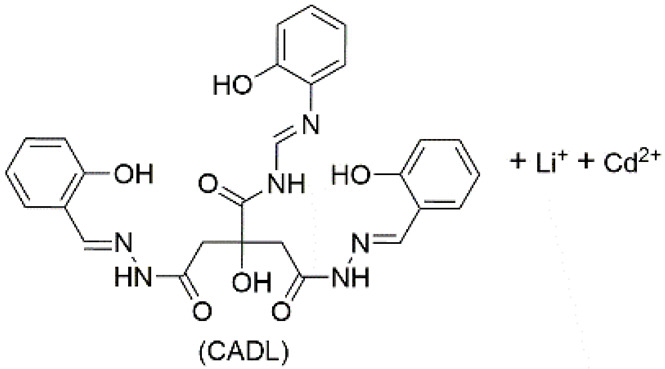	DMF	Li^+^, Cd^2+^, OH^−^, CH_3_COO^−^, CADL	Conductivity (S cm^−1^): 4.06 × 10^−4^ Resistance (Ω): 1724	CADL	378/~475	-	[82]
6	supramolecular bulk hydrogel	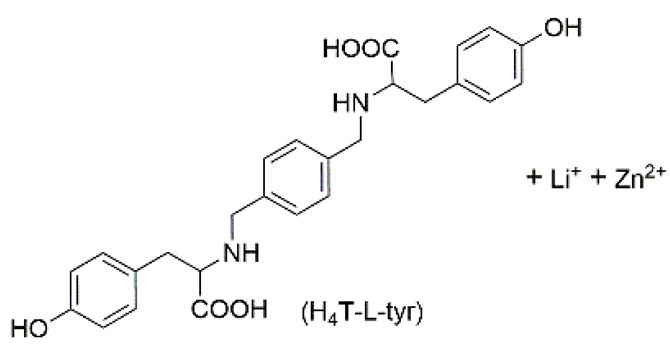	H_2_O	Li^+^, Zn^2+^, OH^−^, NO_3_^−^, H_4_T–L–tyr, Au nanoparticle (NP)	Conductivity (S·cm^−1^): 5.05 × 10^−3^	H_4_T–L–tyr	-/~420	catalysis	[90]
7	supramolecular bulk organogel	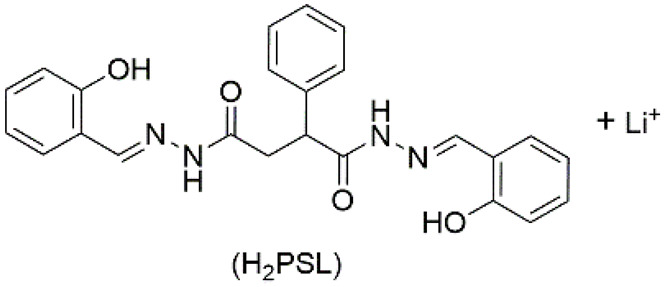	DMF	Li^+^, OH^−^, H_2_PSL	Resistance (Ω): 7.4 × 10^3^	H_2_PSL	320/~472	-	[91]
8	supramolecular bulk hydrogel	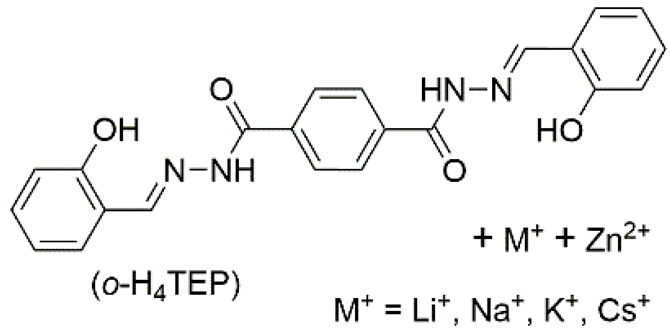	H_2_O	M^+^, Zn^2+^, OH^−^, NO_3_^−^, *o*–H_4_TEP	Conductivity (S cm^−1^): 1.36 × 10^−2^ (MH–Li); 1.44 × 10^−2^ (MH–Na); 1.53 × 10^−2^ (MH–K); 1.60 × 10^−2^ (MH–Cs) Resistance (Ω): 237 (MH–Li); 137(MH–Na); 92 (MH–K); 75 (MH–Cs)	*o*–H_4_TEP	363/~500	-	[92]
9	supramolecular micro-hydrogel	SnO_2_ + chemically converted graphene (CCG)	H_2_O	CCG	Conductivity (S·cm^−1^): 350	rhodamine B	555/~573	DNA detection	[93]
10	supramolecular bulk hydrogel	nitrogen-doped graphene (NG)	H_2_O	NG	-	luminol	-	Escherichia coli sensing	[94]
11	polymeric bulk hydrogel	polyaniline (PAni) + phytic acid (PA)	H_2_O	PAni, PA	-	*N*-(aminobutyl)-*N*-(ethylisoluminol) (ABEI)	-	Live cell H_2_O_2_ detection	[95]
12	polymeric bulk hydrogel	PAni + poly(acrylic acid) (PAA)	H_2_O	PAni, AuNP	Resistance (Ω): 24	luminol, CdTe, quantum dot (QD)	-	cytosensor	[96]
13	supramolecular bulk hydrogel	graphene	H_2_O	graphene, AuNP	-	carbon dot (CD)	410/~517	cytosensor, glucose transporter 4 expression evaluation	[83]
14	polymeric bulk hydrogel	PAni + ATMP	H_2_O	PAni	-	ABEI	-	xanthine detection	[26]
15	polymeric bulk hydrogel	BSA + Au/Ag nanocluster (NC)	H_2_O	Na^+^, K^+^, PO_4_^3−^, Cl^−^, NO_3_^−^	-	Au/Ag NC	500/~620	detection of glutathione (GSH)	[97]
16	supramolecular bulk hydrogel	AuAXP nanocluster (NC) + Ca^2+^	H_2_O	Ca^2+^, Cl^−^	-	AuAXP NC	~500 nm ECL	detection of calmodulin	[98]
17	supramolecular bulk hydrogel	Ag-melamine metal-organic gel (Ag-MOG)	H_2_O	Ag-MOG	-	Tri(2,2’-bipyridyl)dichlororuthenium(II) (Ru(bpy)_3_^2+^);dichlorotris (1,10-phenanthroline) ruthenium (II) (Ru(phen)_3_^2+^)	-	DNA detection	[99]
18	supramolecular bulk hydrogel	Au NP	H_2_O	graphite-like carbon nitride (g-C_3_N_4_), Au NP	-	Au NP	~465 ECL	Zika Virus DNA detection	[100]
19	supramolecular bulk hydrogel	Ag_9_ NC	H_2_O	Ag_9_ NC	Resistance (Ω): 586	Ag_9_ NC	234/~575	methyltransferase Assay	[101]
20	supramolecular bulk hydrogel	tris(4,4′-dicarboxylicacid-2,2′-bipyridyl) ruthenium (II) dichloride (Ru(dcbpy)_3_^2+^) + 4′-(4-carboxyphenyl)-2,2′:6′,2′’-terpyridine (Hcptpy) + Tb^3+^	H_2_O	Tb^3+^, Ru^2+^, Cl^−^, NO_3_^−^	-	Ru(dcbpy)_3_^2+^, Tb complex	-/~608.4; ~679 ECL	epinephrine detection	[102]
21	Supramolecular bulk aerogel	MoS_2_ nanosheet (NS)	air	MoS_2_ NS	Resistance (Ω): 115	polydopamine NP with phenylboronic acid (PBA) (PDA-PBA NP)	-/~457 (PDA-PBA NP); ~600, 562, 552 ECL (PDA-PBA NP)	MiRNA-126 detection	[103]
22	polymeric bulk organogel	poly(ethyl acrylate-*r*-styrene-*r*-divinylbenzene) (PEA-*r*-PS-*r*-PDVB)	1-ethyl-3-methylimidazolium bis(trifluoromethylsulfonyl) imide ([EMI][TFSI])	[EMI][TFSI]	Resistance (Ω): 77,520	Ru(bpy)_3_(PF_6_)_2_	~612 ECL	wearable ionoskin	[104]
23	polymeric bulk organogel	poly(vinylidene fluoride-*co*-hexafluoropropylene) (P(VDF-*co*-HFP))	1-alkyl-3-methylimidazolium bis(trifluoromethylsulfonyl)imide ([AMI][TFSI] including [EMI][TFSI], [BMI][TFSI], [HMI][TFSI], and [DMI][TFSI])	[AMI][TFSI]	Conductivity (S·cm^−1^): 2.5 × 10^−3^ ([EMI][TFSI]); 1.5 × 10^−3^ ([BMI][TFSI]); 0.60 × 10^−3^ ([HMI][TFSI]); 0.28 × 10^−3^ ([DMI][TFSI]) Resistance (Ω): 226 ([EMI][TFSI]) 381 ([BMI][TFSI]) 939 ([HMI][TFSI]) 2018 ([DMI][TFSI])	2,2′-bipyridyl-bis[2-(2′,4′-difluorophenyl)pyridine]-iridium(III) hexafluorophosphate Ir(diFppy)_2_(bpy)PF_6_; Ru(bpy)_3_(PF_6_)_2_	green ECL; red ECL	display material	[105]
24	polymeric bulk organogel	poly(4-vinyl pyridine) (P4VP)	pyridine	P4VP	Resistance (MΩ) 9–10 (after 385 nm radiation)	P4VP	280/~364, 440	-	[106]
25	polymeric bulk xerogel	poly(*N*-[5-(8-hydroxyquinoline)methyl]aniline) (PNQA) + V_2_O_5_	air	PNQA	Conductivity (S·cm^−1^) (after aging): 3.1×10^−3^	PNQA	373/~471	-	[107]
26	polymeric bulk organogel	poly(methyl methacrylate) (PMMA)	1-butyl-3-methylimidazolium bis(trifluoromethane sulfonyl)imide ([Bmim][N(Tf)_2_])	[Bmim][N(Tf)_2_]	Conductivity (S·cm^−1^) 10^−3^	[(Bu’_2_bpy)Pt(C≡CC_6_H_4_tpy)][Eu(hfac)_3_]_2_	310/~616	-	[108]
27	polymeric bulk organogel	poly(*N*-isopropylacrylamide-*co*-*N*-vinylcarbazole) (P(NVC-*co*-NIPA))	dioxane	P(NVC-*co*-NIPA)	Conductivity (S·cm^−1^) (treated by CAN): 0.017 (S1); 0.19 (S2); 0.20 (S3); 0.22 (S4); 0.25 (S5); 0.43 (S6)	P(NVC-*co*-NIPA)	300/- (S1); 300/~373 (S2); 300/~373 (S3); 300/~380, 410 (S4); 300/~380, 410 (S5); 300/~420 (S6)	-	[109]
28	polymeric bulk hydrogel	La-cholate/poly(acrylamimde) double network	H_2_O	La^3+^, Na^+^, CH_3_COO^−^, Cl^−^	Conductivity (S·cm^−1^): 3 × 10^−3^	La complex	-/~430	strain sensor	[110]
29	polymeric bulk organogel	poly(MMA–HEMA)	PC-γ-GBL mixture	TBABF_4_	-	TPETPAOMe BTOTPAOMe	-/~505; -/~551	electrofluorechromic devices	[111]

^a^ network composition of gel; ^b^ some abbreviations of solvents: AN: acetonitrile, EG: ethylene glycol; MeOH: methanol; EtOH: ethanol; PrOH: propanol, *t*-BuOH: *n*-butanol, DMF: *N*,*N*-dimethylformamide, PC: propylene carbonate, γ-GBL: γ-butyrolactone; ^c^ *λ*_ex_: excitation wavelength (for fluorescence), *λ*_em_: emission wavelength at maximum intensity of main band (for fluorescence).

**Table 2:** Summary of information of typical luminescent conductive gel composites involved in this review.

No.	Layer I	Layer II	Layer III	Conductive Species	Electrical Parameter	Luminescent Species	Luminescent Parameter	Application	Ref.
1	polystyrene organic gel + MAPbBr_3_ PNC (luminescent layer)	Ecoflex + ZnS:Cu + BaTiO_3_ (luminescent layer)	PAAm hydrogel + LiCl (ionic conductive layer)	Li^+^, Cl^−^	-	MAPbBr_3_ PNC; ZnS:Cu	*λ*_ex(PL)_/*λ*_em(PL)_: 365/~525 (V-PNC@AINO); EL color: blue (ZnS:Cu-based layer)	soft EL devices	[116]
2	PAA hydrogel + NaCl (ionic conductive layer)	polyurethane + ZnS particle + boron nitride nanosheet (luminescent layer)	PAA hydrogel + NaCl (ionic conductive layer)	Na^+^, Cl^−^	Conductivity (S·cm^−1^): 2 × 10^−3^	ZnS particle	*λ*_em(EL)_: ~450; ~500; ~588	light-emitting device	[117]
3	Ecoflex + ZnS:Cu (luminescent layer)	PAA hydrogel + NaCl (ionic conductive layer)	Ecoflex	Na^+^, Cl^−^	-	ZnS:Cu	*λ*_em(EL)_: ~520	wearable smart skin	[27]
4	agarose-polyacrylamide (PAAm) hydrogel + LiCl (ionic conductive layer)	polydimethylsiloxane (PDMS) elastomer + ZnS:Cu (luminescent layer)	agarose-polyacrylamide hydrogel + LiCl (ionic conductive layer)	Li^+^, Cl^−^	Resistance (Ω): ~20,000	ZnS:Cu	EL color: blue	wearable sensor; flexible EL device	[118]
5	PAAm hydrogel + LiCl (ionic conductive fiber core)	PSPI elastomer + ZnS; PSPI elastomer + CdTe/ZnS QD (luminescent sheath)	-	Li^+^, Cl^−^	Conductivity (S·cm^−1^): 0.16	ZnS; CdTe/ZnS QD	EL color: blue (ZnS-based sheath); PL color: pink (QD-based sheath)	wearable motion sensor	[119]
6	PDMS + carbon nanotube (CNT) (conductive layer)	red: Ru(bpy)_3_(PF_6_)_2_ + [EMI][TFSI] + polymethyl methacrylate (PMMA) + polyethylene glycol (PEG); green: bis-[2-(2,4-difluorophenyl) pyridin ate](2,2′-dimethyl-4,4′-bipyridine)iridium(III) hexafluorophosphate ([Ir-(Fppy)_2_(dmb)]PF_6_) + [EMI][TFSI] + PMMA + PEG; blue: 9,10-diphenylanthracene (DPA) + PMMA + LiCF_3_SO_3_ (luminescent conductive organogel layer)	-	CNT; [EMI][TFSI; LiCF_3_SO_3_	Resistance (Ω·sq^−1^): 805.2–73.5 (CNT solution volume 250–2000 μL) (conductive layer)	Ru(bpy)_3_(PF_6_)_2_, [Ir-(Fppy)_2_(dmb)]PF_6_, DPA	*λ*_em(EL)_: ~616 (red layer); ~532 (green layer); ~430 (blue layer)	wearable sensor	[120]
7	single-walled carbon nanotube (SWNT) (conductive fiber core)	supramolecular organic gel of pyrene-based LMWG (luminescent middle layer) 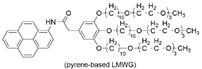	silica wall	SWNT	-	pyrene-based LMWG	*λ*_ex(PL)_/*λ*_em(PL)_: -/~370, 390	-	[121]

## Data Availability

Data is contained within the article.
